# Diet, Probiotics and Their Impact on the Gut Microbiota during the COVID-19 Pandemic

**DOI:** 10.3390/nu13093172

**Published:** 2021-09-11

**Authors:** Marzena Jabczyk, Justyna Nowak, Bartosz Hudzik, Barbara Zubelewicz-Szkodzińska

**Affiliations:** 1 Department of Nutrition-Related Disease Prevention, Faculty of Health Sciences in Bytom, Medical University of Silesia, Piekarska 18 Street, 41-902 Bytom, Poland; marzena.jabczyk@gmail.com (M.J.); bzubelewicz-szkodzinska@sum.edu.pl (B.Z.-S.); 2 Department of Cardiovascular Disease Prevention, Faculty of Health Sciences in Bytom, Medical University of Silesia, Piekarska 18 Street, 41-902 Bytom, Poland; bartekh@mp.pl; 3 Silesian Center for Heart Diseases, Third Department of Cardiology, Faculty of Medical Science in Zabrze, Medical University of Silesia, 41-800 Zabrze, Poland

**Keywords:** diet, microbiota, pandemic, COVID-19, SARS-CoV-2

## Abstract

SARS-CoV-2 infection is associated with diverse clinical manifestations, immune dysfunction, and gut microbiota alterations. The nutritional and biochemical quality of one’s diet can influence the intestinal microbiota, which may play a role in the defense mechanisms against potential pathogens, by promoting a wide variety of immune–host interactions. In the COVID-19 pandemic, besides the development of pharmacological therapies, a healthy balanced diet, rich with food-derived antioxidants, may be a useful strategy. Many studies demonstrated that vitamins and probiotic therapies have positive effects on the treatment and prevention of oxidative stress and inflammation in COVID-19. The ecology of the gut microbiota in the digestive tract has been linked to the transport function of the host receptor known as angiotensin converting enzyme 2 (ACE2), suggesting that COVID-19 may be related to the gut microbiota. The angiotensin converting enzyme (ACE), and its receptor (ACE2), play central roles in modulating the renin–angiotensin system (RAS). In addition, ACE2 has functions that act independently of the RAS. ACE2 is the receptor for the SARS coronavirus, and ACE2 is essential for the expression of neutral amino acid transporters in the gut. In this context, ACE2 modulates innate immunity and influences the composition of the gut microbiota. Malnutrition is one of the leading underlying causes of morbidity and mortality worldwide and, including comorbidities, may be a major cause of worse outcomes and higher mortality among COVID-19 patients. This paper reviews the research on dietary components, with particular emphasis on vitamins, antioxidants, and probiotic therapies, and their impacts on the intestinal microbiota’s diversity during the SARS-CoV-2 pandemic.

## 1. Introduction

The gastrointestinal (GI) tract is considered to be the largest immune organ of the body and harbors an intricate community of commensal microorganisms [1,2]. The gut microbiome contains approximately 160 species of bacteria, although this number can vary based on environmental and genetic factors. In the gut, *Bacteroidetes* and *Firmicutes* are predominant, while in the lung, *Proteobacteria* are also present. Approximately 70–80% of the body’s immune cells are located in the GI tract, indicating a significant connection between the immune system and intestinal microflora [1]. Disrupted gut microbiota can result in elevated levels of angiotensin-converting enzyme 2 (ACE2), due to epithelium breakdown and inflammation. ACE2 was reported to be a key target of the SARS-CoV-2 virus by triggering the inflammatory cascades (“cytokine storm”) known to worsen the severity of the COVID-19 disease [3]. The angiotensin converting enzyme (ACE) and its receptor (ACE2) play central roles in modulating the renin–angiotensin system (RAS). In addition, ACE2 has functions that act independently of the RAS. ACE2 is the receptor for the SARS coronavirus, and ACE2 is essential for the expression of neutral amino acid transporters in the gut. In this context, ACE2 modulates innate immunity and influences the composition of the gut microbiota [4].

Although the GI tract is one of the major players in fluid and electrolyte intake and excretion, the role of RAS in the GI system was only recently well-characterized. There is, now, consistent preclinical evidence indicating the presence of all RAS components (including ACE2) required for autonomous regulation throughout the GI tract, supporting their involvement in GI physiology and pathophysiology [5]. An association between gut microbiota dysbiosis and the poor outcomes among elderly COVID-19 patients, particularly among those with pre-existing cardiovascular, cardiometabolic, and cardiorenal diseases, can be hypothesized based on two factors: (1) the link between age-related gut microbiota dysbiosis and cardiometabolic, cardiorenal, and inflammatory disease and (2) the link between gut microbiota dysbiosis and inaccurate local/distal host immunity towards viral infection and RAS deregulation, driven by SARS-CoV-2-induced ACE2 shedding. Indeed, strong epidemiological and biochemical data from the COVID-19 pandemic show that elderly people with pre-existing cardiovascular, metabolic, renal, and lung diseases (including hypertension, coronary disease, diabetes, CKD, and respiratory syndromes) are at higher risk of severe disease and mortality when infected with SARS-CoV-2 [4]. Hu J et al. [2] reported a poor prognosis among COVID-19 patients with underlying comorbidities that produce elevated gut permeability and diverse lower gut microbiota. Diet and probiotic therapy are essential to support the gut microbiome balance and may, therefore, affect the microbiome’s diversity [2]. The development of pharmacological therapies and vaccinations in the COVID-19 pandemic has played a significant role in the prevention and treatment of the SARS-CoV-2 infection. Nevertheless, the use of dietary supplements may be another useful strategy [6]. Simopoulos A.P [7] suggested that the genomic variety of the microbiome, and its possible interactions with the diets in various populations, should be taken into consideration when considering the possible factors that may contribute to health disparities in the development of COVID-19. A healthy diet with a balanced omega-6 and omega-3 fatty acid ratio is essential to mitigate a chronic low-inflammatory state and, thereby, allow one to maintain normal immune-system reactions. These actions, as a consequence, would decrease the risk of contracting the SARS-CoV-2 virus [7]. Moreover, malnutrition is one of the most relevant factors leading to higher morbidity and mortality in chronic and acute disease following infection with SARS-CoV-2 [8]. Preventive, diagnostic, and therapeutic interventions in malnutrition should be included in the management strategies for COVID-19 patients, specifically among older adults and those who require intensive care [8]. For this reason, the European Society for Clinical Nutrition and Metabolism (ESPEN) published practical guidelines for the nutritional management of COVID-19 patients.

This paper reviews the research in the context of dietary components, with particular emphasis on vitamins, antioxidants, and probiotic therapies, and their impacts on intestinal microbiota diversity in the SARS-CoV-2 pandemic.

## 2. Gut Microbiome Alterations and COVID-19 Disease Severity

Moreira-Rosario et al. [9] investigated the influence of changes in the gut microbiota composition on COVID-19 severity. The results of the study revealed a change in the Firmicute and Bacteroide ratio, which the authors classified as a hallmark of dysbiosis. Moreover, the authors observed a decreased abundance of butyrate-producing bacteria from the family Lachnospiraceae (in particular, *Reseburia* and *Lachnospira*), a lower abundance of bacteria from the Actinobacteria phylum (including *Bificobacteria* and *Collinsella*), and an increased abundance of Proteobacteria in moderate and severe COVID-19 patients, compared to mild COVID-19 patients. Yeoh et al. [10] further highlighted that the associations between gut microbiota composition, cytokine levels, and inflammatory markers may be involved in determining the magnitude of SARS-CoV-2 infection severity. The authors investigated whether changes in the gut microbiome of SARS-CoV-2-infected patients would resolve after clearance of the SARS-CoV-2 virus, and if microbiome alterations were linked to disease severity. The authors determined that the composition of the gut microbiota was significantly different in COVID-19 patients, compared to those in the non-COVID-19 group (*p* < 0.01). The composition of the gut microbiota in SARS-CoV-2-infected patients exhibited a greater relative abundance of *Bacteroidetes*, contrary to the control group, whereas *Actinobacteria* were more common in non-COVID-19 patients (*p* < 0.05). *Faecalibacterium prausnitzi* (an anti-inflammatory bacterium) and *Bifidobacterium bifidum*, which are known to play immunomodulatory roles in the gastrointestinal tract, were negatively correlated with COVID-19 severity, based on a correlation with antibiotic use and patient age. In turn, two other species, *Akkermansia muciniphila* and *Bacteroides dorei*, were positively correlated with IL-1β and IL-6 levels. Similar results were found in a pilot study by Zuo et al. [11], who observed the depletion of commensal symbionts, including the *F. prausnitzi*, *Roseburia*, *Eubacterium ventriosum*, and *Lachnospiraceae* taxa in the gut, and the enrichment of opportunistic pathogens, including *Bacteroides nordii*, *Clostridium hathewayi*, and *Actinomyces viscosus*. The researchers also noted a relationship between the abundance of the bacteria *C. hathewayi* and COVID-19 severity.

## 3. Diet and Probiotics in SARS-CoV-2 infection

Weight loss may be an alternative explanation for the changes in the content of the gut microbiome described in respiratory infections. Diet is the primary factor contributing to the composition of the gut microbiota. A reduction in caloric intake among humans is associated with significant increases in *Bacteroidetes* compared to *Firmicutes*. Caloric restrictions in combination with infection by the influenza virus were shown to exacerbate the alterations in the intestinal microbiome observed in influenza alone [12,13]. Most studies on human and mouse models aimed to improve the immune response and relieve influenza virus infection using probiotic therapies, mainly through therapy with *Lactobacillus* spp. [14]. Enrichment of the gut microbiota with *Lactobacillus* may protect against respiratory tract inflammation in RSV infections or influenza [12].

Patients with mild or asymptomatic COVID-19 infections, as well as subjects in quarantine, are recommended to follow a healthy, balanced, and anti-inflammatory diet, rich in whole grains, legumes, vegetables, and fruits [15]. Many studies have noted an inverse correlation between dietary fiber intake and the serum levels of inflammatory markers, including C-reactive protein, interleukin-6 (IL-6), interleukin-18 (IL-18), and tumor necrosis factor alpha (TNFα). Moreover, high-fiber diets are linked to lower glycemia and higher plasma concentrations of adiponectin, which are beneficial for the anti-inflammatory effect mediated by the insulin-sensitizing activity of adipocytokines. Conte and Toraldo [15] reported that the anti-inflammatory properties of dietary fiber may strongly support the activities of antiviral and immunosuppressive drugs. Ratajczak et al. [16] also confirmed the beneficial effects of complex carbohydrates and dietary fiber on the fermentation of some species of gut microbiota via the production of metabolites, including SCFAs (mainly acetate, propionate, and butyrate), which regulate host metabolism by lowering colonic pH and positively affecting the serum lipid profile, thereby enhancing the immune response far beyond the gut [17], the immune system itself (including the lungs), and cell proliferation [16]. In addition, the consumption of whole grain components and dietary fiber levels were reported to have beneficial effects on pulmonary diseases and to reduce mortality from chronic respiratory diseases [18]. This result may be explained by in vivo studies suggesting that whole grains that are high in fiber are also rich in the phenolic compounds able to promote the growth of potentially beneficial organisms (such as *Lactobacillus*, *Bifidobacterium*, *Akkermansia*, and *Faecalibacterium*) while inhibiting *Helicobacter pylori* and *Staphylococcus species* [17]. Fat consumption may also have potential benefits in terms of gut microbiota composition. A study published in the *Indian Journal of Microbiology* observed a higher death rate from COVID-19 in Western countries compared to that in India, which can be attributed to several factors. The Western diet is richer in saturated fatty acids, which exacerbate intestinal dysbiosis, thereby causing a cytokine storm. Moreover, the greater exposure to infections in India compared to that in developed nations resulted in a better-prepared immune response against a wider range of microbes [17]. Notably, a diet rich in animal protein and saturated fats reduces the beneficial phyla in the human gastrointestinal tract, including *Bifidobacterium* or *Faecalibacterium*, while unsaturated fats reduce harmful microbes, such as *Escherichia* and *Streptococcus* [19].

Scarpellini et al. [20] analyzed the interactions of the gut microbiota and the liver mediated by the immune system during the SARS-CoV-2 pandemic, and detected the immunosuppressive effects of alcohol consumption. Ethanol inhibits the NK cell response and depletes other types of lymphoid cells. These effects are associated with intestinal dysbiosis and increase susceptibility to infections and viral diseases.

A number of polyphenols demonstrated anti-inflammatory effects by influencing cytokine secretion, thereby promoting the expression of proinflammatory genes [21,22]. In addition, these polyphenols showed antiviral activities against several viruses [23]. Curcumin is a polyphenol that can be used as a potential support in the treatment of patients with COVID-19 [24,25]. Utomo et al. [26] showed that curcumin can bind to the target receptors of SARS-CoV-2 protease (receptor binding domain (RBD)) and the ACE2 protease domain to support the prevention of SARS-CoV-2 infections. In addition, a combination of three ingredients—vitamin C, curcumin, and glycyrrhizic acid (a major phytonutrient of licorice root)—promotes the synthesis and release of interferons and, thus, plays an important role in regulating the inflammatory process. This phenomenon suggests that a combination of these three phytonutrients may be helpful in regulating the immune response against SARS-CoV-2 infection [25,27].

To date, the roles of vitamin C supplementation in the prevention of SARS-CoV-2 infection, and as a supportive treatment for COVID-19, are not fully understood, as the results are conflicting [25,28]. However, given the lack of a specific therapy for COVID-19, vitamin C may play some role in the respiratory viral infection [28]. Several randomized trials have demonstrated a positive effect of vitamin C supplementation on the symptoms and duration of respiratory infections, and some data also suggest that vitamin C can prevent pneumonia and alleviate the course of other infections [18,29]. Moreover, Yang et al. [19] demonstrated that supplementation with vitamin C (the best nutritional sources of vitamin C include lemon, spinach, and broccoli), vitamin D (fish and eggs), and vitamin E (almonds, spinach, broccoli, and olive oil) facilitates a beneficial microbiome (increasing the abundance of *Bifidobacterium*, *Lactobacillus*, and *Roseburia* microorganisms) and reduces the ratio of *Firmicutes* to *Bacteroidetes* in the microbiota composition, thereby yielding favorable effects [17].

The potential impact of vitamin D supplementation on reducing the risk of respiratory infections, including COVID-19, is also of interest. Grant et al. [30] identified several mechanisms through which vitamin D supplementation could help reduce the risk of infection. These mechanisms include the induction of cathelicidin and defensin synthesis, which can reduce the rate of viral replication, lower the concentration of proinflammatory cytokines, and increase the concentration of anti-inflammatory cytokines. Vitamin D enhances the synthesis of antimicrobial peptides in the epithelium of the respiratory tract. In this way, there are several pathophysiological mechanisms indicating that vitamin D could mitigate the chance of SARS-CoV-2 infection and COVID-19 symptoms. The subsequently generated 25(OH)2D interacts with a protein in the renin–angiotensin (ACE2) system used by SARS-CoV-2 as an exit receptor. The presence of the virus lowers ACE2 expression. However, vitamin D promotes the expression of the ACE2 gene [31].

Many molecular mechanisms are involved in the effects of vitamin D, which can modulate the immune response, decrease the risk of infection, and potentially provide a beneficial balance in the inflammatory response [30]. The vitamin D receptor is present in the epithelial cells of the respiratory tract, and the macrophages in the respiratory system. Moreover, the 25-hydroxylase that converts vitamin D into its active metabolite is present in the epithelium of the respiratory tract. An in vitro study demonstrated that 1,25(OH)2D3 increases the synthesis of cathelicidin by macrophages. This peptide can bind to the envelope of influenza A and respiratory syncytial viruses to damage the envelope structures and prevent infections [32]. A previous analysis evaluated 14,108 patients (>16 years of age) and the associations between the probability of developing an acute respiratory infection and several limiting factors, such as season, demographics, and clinical data. The results demonstrated that vitamin D levels of <30 ng/mL were associated with a 58% higher risk of acute infection compared to subjects with vitamin D concentrations of ≥30 ng/mL [33]. A meta-analysis of 25 randomized controlled trials involving 10,933 participants showed that vitamin D diminished the risk of acute respiratory infections [34]. 

Other studies have suggested the potential effectiveness of vitamin D supplementation in the prevention and treatment of COVID-19. Vitamin D deficiency was suggested to result in elevated susceptibility to some respiratory virus infections, particularly respiratory syncytial virus (RSV) and influenza infection [35].

Vitamin D also induces a nonspecific response that can lead to the general inhibition of viral infection. Following the initiation of infection, vitamin D may modulate the process through interactions with both cellular and viral factors (via the induction of autophagy and apoptosis). Vitamin D supplementation may play a prominent role in infection through the inhibition of inflammatory reactions, an increase in ACE2 expression, a decrease in the neutrophil to lymphocyte ratio, and the inhibition of the complement. However, there is an inconsistency in this hypothesis. The overexpression of ACE2 may also increase the risk of severe COVID-19, due to its negative regulatory impact on the renin–angiotensin system and, as a consequence, increase the chance that the virus will bind to the host cells [36]. Therefore, there seems to be a definite need to evaluate the optimal dosage and time of vitamin D supplementation as a therapeutic approach.

An Italian research group proposed a nutritional protocol for COVID-19 patients that includes vitamin D supplementation in the case of vitamin D deficiency [37].

Active forms of vitamin A include retinal, retinol, and retinoic acid. Retinoic acid plays a key role in modulating the differentiation, maturation, and function of the innate immune system (e.g., macrophages [38] and neutrophils [39]), and promotes an immediate response to pathogen invasion through phagocytosis and the activation of cytotoxic T cells [40]. On the other hand, Iddir et al. [40] indicated that a systematic study among children failed to demonstrate any effect of vitamin A supplementation on the risk of lower respiratory tract diseases or symptoms, according to a meta-analysis of previous studies.

## 4. Malnutrition in SARS-CoV-2 Infection

**Figure 1:**
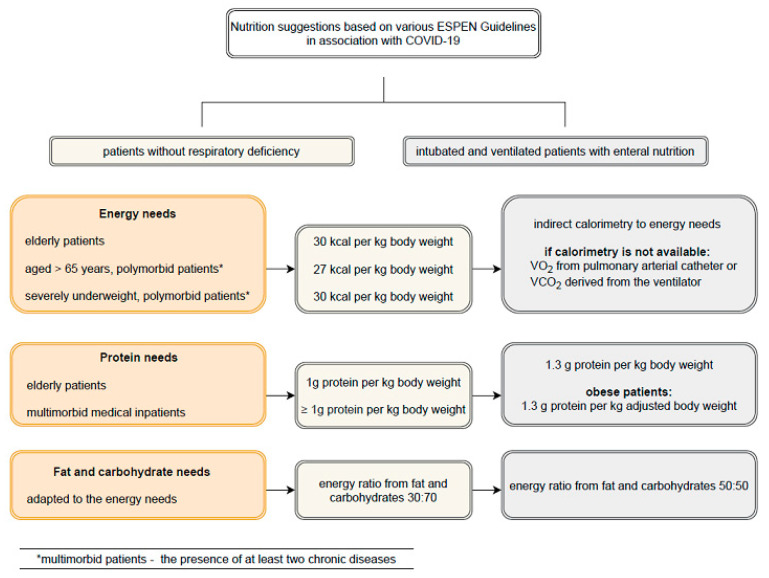
Nutritional suggestions based on the ESPEN Guidelines in association with COVID-19 [8].

As noted previously, preventive, diagnostic, and therapeutic interventions in malnutrition may play a role in the prognosis of COVID-19 patients. A cross-sectional study on nutritional assessment and therapy among SARS-CoV-2-infected hospitalized patients in Italy showed that 77.2% of the patients suffered from a high prevalence of nutritional risk, and half (49.7%) were malnourished [41]. In ESPEN, expert statements and practical guidance for the nutritional management of patients with SARS-CoV-2 infection were used to propose recommendations for malnourished individuals. The authors highlighted checking for malnutrition through screening and assessments; discussing the challenges and strategies for optimizing nutritional status; receiving counseling from experienced professionals; and supplementation with vitamins A and D, and other micronutrients. Conversely, the authors noted that older obese individuals with chronic diseases should also be included in the recommendations, due to the risk of reduced skeletal muscle mass and function [8]. The dedicated energy needs, protein needs, and carbohydrate and fat needs are presented in Figure 1 [8].

## 5. Probiotic Therapy

Probiotic strains may affect type I interferon levels and increase the number and activity of antigen-presenting cells, NK cells, and T cells, as well as the levels of systematic and mucosal-specific antibodies in the lung [42]. Supplementation with probiotics in animal models also promoted the proliferation of *Bifidobacterium* and increased the production of SCFAs [43]. The exact mechanism underlying the antiviral action of probiotics is not entirely clear, and according to Olaimat et al. [44], the effects likely involve several mechanisms, including the following:

1. Strengthening of the innate immune responses of the mucosa;
2. Decreasing intestinal permeability;
3. The effects on the systemically acquired immune response are mediated by regulatory and anti-inflammatory effects.

The potential of probiotic therapy in reducing the risk and severity of respiratory viral infections was confirmed by clinical and experimental studies of influenza, rhinovirus, and respiratory syncytial virus. Probiotics have not been tested in SARS-CoV-2 therapy, and it should be noted that some bacterial strains demonstrated antiviral activity against other coronaviruses [42]. Two meta-analyses demonstrated the effectiveness of probiotics in reducing the prevalence and duration of viral respiratory tract infections [45,46]. Mak et al. [47] interpreted the results of a study carried out by Hao et al. [46] as moderate (the number of cases was reduced twofold), while Giannoni et al. [48] emphasized that the results of the study indicated considerably higher effectiveness of probiotic therapy than “moderate”. Two other randomized controlled trials demonstrated that critically ill and mechanically ventilated patients who were given probiotics (*Lactobacillus rhamnosus GG*, live *Bacillus subtilis*, and *Enterococcus faecalis*) developed pneumonia less frequently, compared to patients treated with a placebo [49,50]. Although a series of Chinese cases revealed that some COVID-19 patients presented intestinal dysbiosis with a reduced abundance of *Lactobacillus* and *Bifidobacterium* [51], animal studies showed that *Lactobacillus acidophilus* and *Bacillus clausii* did not reduce coronavirus receptor expression in the mouse small intestine, compared to that of the control models or groups, after *Salmonella* infection [52]. *Lactobacillus* spp. and *Bifidobacterium* spp. are two types of nonpathogenic bacteria. Thus, a detailed investigation of the pathogenesis of SARS-CoV-2, and its impact on the gut microbiota, is needed before these probiotics can be recommended for COVID-19 patients [47]. However, the safety of probiotics, not only among the most vulnerable populations (including those in intensive care units), but also among subjects with mild COVID-19, or in preventing infection, may be questionable. According to Baud et al. [42], probiotics are generally safe, even among the most vulnerable groups that are at higher risk. This assumption was confirmed by studies conducted on premature infants [53,54]. Moreover, to flatten the infection curve for COVID-19, the use of probiotics and prebiotics (e.g., fructans and galactans) was recommended to enhance the propagation of probiotic strains and native beneficial microbes [42]. Accordingly, the results of a meta-analysis of randomized controlled trials carried out by Chan et al. [55] indicated the prevention of respiratory tract infections via symbiotic interventions.

Interestingly, the National Health Commission of China has recommended the use of probiotics to treat patients with severe COVID-19, to maintain gut balance and prevent secondary infections [56,57]. However, the treatment of COVID-19 includes antibiotics and antiviral drugs [56], which can lead to a risk of diarrhea. Therefore, probiotic therapy may be justified, not only due to its therapeutic potential, but also as a means of intestinal prophylaxis after treatment with antibiotic therapy.

Saleh et al. [58] concluded that the introduction of probiotics and/or prebiotics should be considered during the treatment and development of a strategy for managing the pathogenesis of COVID-19. The purpose of probiotic therapy is to restore intestinal homeostasis, limit inflammation, exacerbate the immune response, and prevent mitochondrial stress.

An interesting hypothesis was suggested by *Belojevic* and *Prasher* [59], who proposed the use of at least 109 colony-forming units of *Lactobacillus* spp. or *Bifidobacterium* during a meal, for prophylactic purposes, once a day and up to three times a day, among subjects infected with COVID-19. Notably, the authors did not receive financial support for this study, and the publication was not a form of advertising for any probiotic preparation.

Supplementation with prebiotics that support probiotic therapy is one of the methods used to modulate the gut microbiome. Prebiotics are classified as substances resistant to low pH gastric juices that are hydrolyzed by digestive enzymes and not absorbed or fermented in the intestinal lumen. Moreover, prebiotics stimulate the activity of the gastrointestinal tract by providing a partial supply of the substrates necessary for the production of metabolic products by the gut microbiota [44]. Group of compounds showing potential beneficial effects in the prevention and treatment of SARS-CoV-2 infection are showed in Table 1.

## 6. Conclusions

The interaction between the gut microbiota and COVID-19 was reported in several papers. Diet, probiotics, and prebiotics are the primary modulators in maintaining gut biodiversity and supporting the immune response. Nutritional strategies directed toward restoring a beneficial gut microbiome may help suppress viral infection in the elderly and those with comorbidities.

Furthermore, large-scale international randomized clinical trials are needed to find a link between dietary probiotic therapies, the gut microbiome, susceptibility to SARS-CoV-2 infection, and the severity of COVID-19. These studies should focus on clinical data, dietary patterns, genetic associations, and environmental exposure. 

## Figures and Tables

**Table 1:** Group of compounds showing potential beneficial effects in the prevention and treatment of SARS-CoV-2 infection.

Group of Compounds	Therapeutic Effects	References
Curcumin	Binding to the SARS-CoV-2 target receptors	[25,26]
Polyphenols	Modulation of cytokine production and the expression of proinflammatory genes. Antiviral activities against several viruses (SARS-CoV-2 has not been proven)	[21,23]
Vitamin C	Alleviating the course of upper respiratory tract infections. Participation in the processes of phagocytosis and chemotaxis. Participation in the production, differentiation, and proliferation of T cells (resulting in the synthesis of antibodies)	[24,25,60]
Vitamin D	Participation in the macrophage synthesis of cathelicidin. Regulation of the activity and levels of NF-kB, IL-6, IL1-β, and TNF-α, and the production of GM-CSF, IL-4, IL-5, VCAM-1, ICAM-1, and E-selectin. An adequate supply of 25(OH)D_3_ protects against acute respiratory infections	[24,25,30,31,32,33,34,37]
Combination of vitamin C, curcumin, and licoric acid	The production of interferons and the regulation of inflammatory responses	[25,27]
Probiotics	*Bifidobacterium* and *Lactobacillus* have beneficial effects against infections (e.g., the influenza virus), including an increase in the number of helper T cells in the lung parenchyma. *Lactobacillus plantarum* and *Lactobacillus reuteri* reduce the recruitment of granulocytes and the expression of the proinflammatory cytokines that inhibit the development of pneumonia virus. *Lactobacillus rhamnosus* increases interferon-γ and interleukin-2. *Bifidobacterium infantis* inhibits IL-17. *Bifidobacterium animalis* prevents virus replication. *Bifidobacterium lactis* increases the proportions of total, helper (CD4^+^), and activated (CD25^+^) T cells, and NK cells in the blood. *Lactococcus lactis* activates plasmacytoid dendritic cells. *Lactobacillus plantarum* reduces the tissue damage caused by inflammation in TGEV (gastroenteritis coronavirus). *Bacillus subtilis* inhibits the adherence of TGEV	[14,18,61,62]
Prebiotics	Inulin decreases the abundance of *Bifidobacterium*, *Lactobacillus*, and *Eubacteria*. Oligofructose decreases the number of bacteria of the *Clostridium*, *Bacteroides*, and *Fusobacterium* genera	[63]
